# Subgrain-controlled grain growth in the laser-melted 316 L promoting strength at high temperatures

**DOI:** 10.1098/rsos.172394

**Published:** 2018-05-09

**Authors:** Kamran Saeidi, Farid Akhtar

**Affiliations:** Department of Mathematics and Engineering Sciences, Division of Engineering Materials, Luleå University of Technology, 97187 Luleå, Sweden

**Keywords:** selective laser melting, thermal treatment, grain growth, mechanical properties

## Abstract

Stainless steel 316 L prepared by laser melting consisted of a hierarchical austenitic microstructure with micrometre-sized (10–25 µm) grains containing fine 1 µm subgrains with a cellular structure. At high-temperature thermal treatments (greater than or equal to 1100°C), merging and growth of the 1 µm subgrains into bigger subgrains restricted the rapid grain growth and microstructure coarsening. Partial phase transformation of austenite to ferrite at temperatures greater than or equal to 1100°C, in combination with gradual and steady growth of subgrains inside the micrometre-sized grains and nucleation of the sigma phase, has promoted the tensile strength of stainless steel 316 L to 300 MPa at 1100°C compared with that of conventionally made 316 L counterparts (approx. 40 MPa). The grain growth mechanism of the laser-melted microstructure can change the application criteria for 316 L and expand the application fields for 316 L.

## 1. Introduction

The microstructure of a polycrystalline material is a key parameter in determining a wide range of its properties, including mechanical strength, toughness, electrical conductivity and magnetic susceptibility [1–3]. One important aspect of the microstructure is the size of the grains and its influence on the properties. Thus, in order to design the grain size and restrict grain growth, microstructural engineering has become of fundamental importance [4]. The boundary between one grain and its neighbours (grain boundary) is a defect in the crystal structure and is associated with a certain amount of energy. The grain growth is a thermodynamically favourable process as it results in a reduction in the total grain boundary energy of the polycrystalline material [5]. Grain/subgrain growth occurs under two mechanisms: (i) movement of the grain boundaries and enlargement of the growing grains at the expense of others and (ii) merging of subgrains with low-angle grain boundaries (LAGBs) into bigger subgrains. The subgrains can merge and grow larger because of annihilation (dispersion) of the LAGBs separating them [6]. High-temperature applications are critically influenced by the grain growth, precipitation and phase changes. Abnormal grain growth, where one or more grains will grow abnormally in the microstructure, occurs in metals at high temperatures. The presence of abnormally large grains in the microstructure may be detrimental to the mechanical properties of polycrystalline materials [7]. The microstructure of laser-melted materials consists of multi-length-scale microstructural features (hierarchies) from micrometre-sized melt pools down to a submicrometre cellular substructure. In this research, the grain growth mechanism by subgrain merging in the laser-melted 316 L stainless steel, after being subjected to high temperatures, is reported that, in turn, helps to avoid abnormal grain growth and microstructure coarsening. In addition, the effects of such a mechanism and phenomenon are consequently seen on the high-temperature mechanical strength and compared with conventional 316 L.

## 2. Material and methods

The pre-thermally treated samples were made by selective laser melting (SLM) in an EOSINT M270 laser sintering device (EOS GmbH, Krailing, Germany) from 316 L powder (Carpenter Powder Products AB, Torshalla, Sweden) and had a composition of 12.2 wt% Ni, 17.6 wt% Cr, 2.31 wt% Mo, 0.45 wt% Si, 0.01 wt% C and Fe. Five samples were built with dimensions of 10 mm × 10 mm × 5 mm. The processing parameters were set to a power of 190 W, a layer thickness of 20 µm and a scan speed of 700 mm s^−1^, and were detached from the building plate using electrical discharge machining. The laser-melted samples were thermally treated at 1000°C, 1100°C, 1200°C and 1400°C for 1 h under an Ar atmosphere and furnace cooled. X-ray diffraction (XRD) analysis was carried out at room temperature in a PANalytical XPert Pro diffractometer using a CuK*α* radiation source. Samples were electro-etched in a 20% NaOH solution at 3 V with a Struers LectroPol-5 device (Struers, Ballerup, Denmark). Microscopy was performed with a Nikon optical microscope (ECLIPSE MA200, Tokyo, Japan) and a scanning electron microscope (SEM) (JEOL, JSM-IT300LV). High-temperature tensile tests at 1100°C and 1200°C were carried out in a Gleeble-3800 thermal–mechanical simulator (DSI, New York, USA) with a strain rate of 0.05 S^−1^ under vacuum.

## 3. Results and discussion

**Figure 1.**
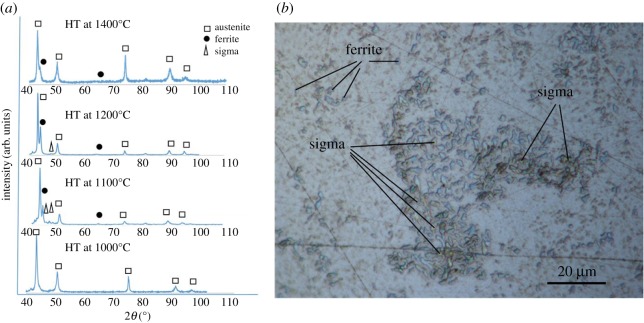
(*a*) Room temperature XRD of SLM 316 L material heat treated (HT) at 1400°C, 1200°C, 1100°C and 1000°C. (*b*) An optical image showing ferrite (blue colour) and the sigma phase (orange/brown colour), also shown by the arrows in SLM heated to 1100°C.

XRD phase identification of laser-melted 316 L thermally treated at 1000°C, 1100°C, 1200°C and 1400°C is depicted in figure 1*a*. This figure shows the presence of a single austenite phase up to 1100°C and partial phase transformation from austenite to ferrite at temperatures greater than or equal to 1100°C. Moreover, the low-intensity diffraction peaks of the sigma phase are observed at 1100°C and 1200°C. The optical micrograph in figure 1*b* shows the sigma phase (orange/brown colour) and ferrite (blue colour), which are depicted by arrows for the specimen heat treated at 1100°C (figure 1*b*). It has been reported previously [8] that ferrite formation could be promoted, on the one hand, by the presence of trace amounts of ferrite nuclei in the as-built SLM 316 L and, on other hand, by the redistribution of Mo, a ferrite stabilizer, in the microstructure of the as-built SLM 316 L.

**Figure 2.**
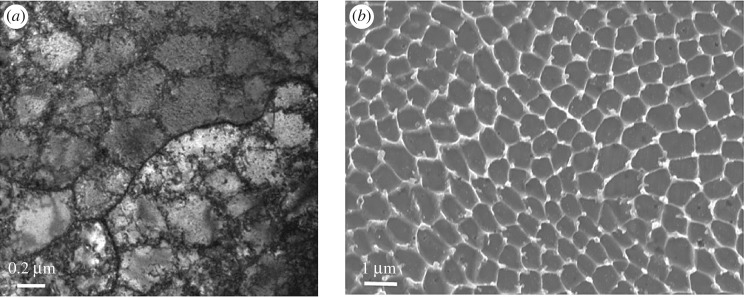
(*a*) Bright-field TEM image showing the dislocation cells and (*b*) SEM image showing the cellular substructure (subgrains).

As-built SLM 316 L has a singular austenitic phase proved by XRD and phase mapping [9,10]. The microstructure of SLM 316 L is hierarchical where the 1 µm sized cellular substructure piled up with dislocations (dislocation cells) is confined inside macro-sized grains (10–25 µm). The dislocation cells are shown in a bright-field transmission electron microscopy (TEM) image in figure 2*a*. The size corresponds to the size of the cellular substructure shown in figure 2*b*. Detailed characterization, carried out by Saeidi *et al*. [11] using scanning transmission electron microscopy (STEM) and TEM mapping techniques, reported that the substructure boundaries were formed by the segregation of Cr and Mo. It has been widely reported that the cellular substructure is actually cellular subgrains with LAGBs [12–15]. Wang *et al*. [12] verified the cellular subgrain structure in laser powder bed fusion (L-PBF) 316 L with LAGBs by point-to-point misorientation measurements and revealed a misorientation angle of 1–2° between the cells. In addition, a kernel average misorientation map performed by Wang *et al*. [12] revealed an up to 1.5° misorientation inside the cells. Niendorf *et al*. [16] verified that the misorientation between the subgrains is relatively small (approximately 2°).

**Figure 3.**
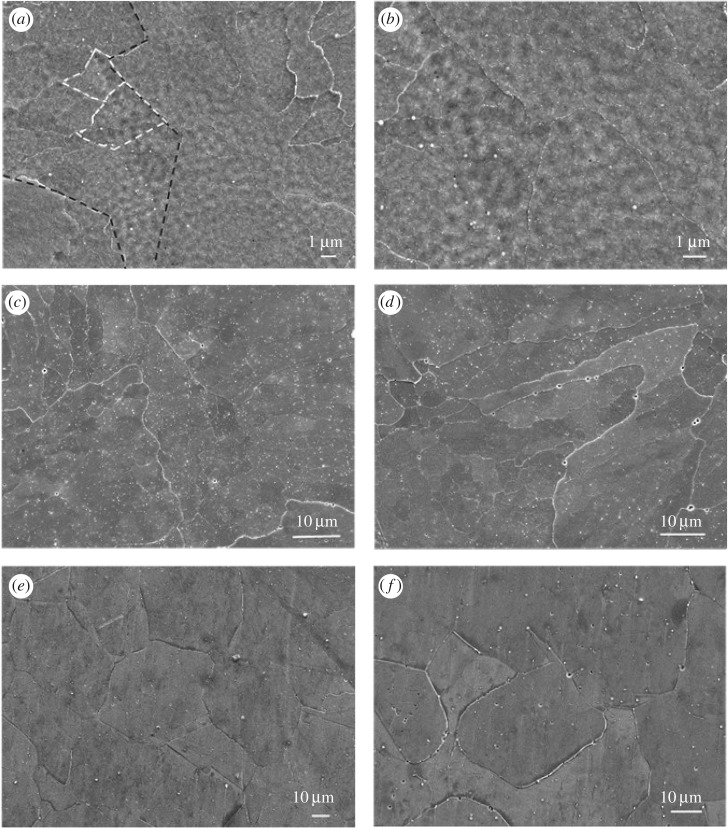
SEM image of samples treated at (*a*,*b*) 1100°C, (*c*,*d*) 1200°C and (*e*,*f*) 1400°C.

A clear tendency of subgrains merging into bigger subgrains is seen in figure 3*a*,*b*. The white dashed areas show how the 1 µm sized cells merge and become a bigger subgrain (approx. 2–5 µm) and the black line marks the boundary of the initial macro-sized grain (approx. 10–20 µm) containing the 1 µm subgrains. By applying a higher temperature (1200°C), it seems that the subgrains have disappeared and the irregular macro-sized grains have remained (figure 3*c*,*d*). At 1400°C, grain growth is observed and the grains grow much larger, with size approximately 30–50 µm. Note that abnormal grain growth has occurred in some areas (figure 3*e*,*f*).

**Figure 4.**
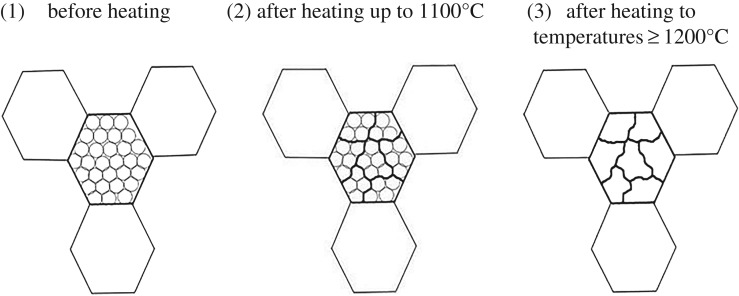
Schematic illustration of the growth mechanism shown in three steps: (1) before heating, (2) heating up to 1100°C and (3) after heating to temperatures greater than or equal to 1200°C.

The mechanism of growth experienced in heat-treated laser-melted 316 L samples can be seen schematically in figure 4. In step 1, the cellular subgrains inside and confined by the macro-grains (shown by the hexagon) are seen. During heating at temperatures up to 1100°C (step 2), the subgrain boundaries merge with adjacent subgrains and form larger subgrains while still being confined in large macro-grains. In step 3, upon heating to a temperature greater than or equal to 1200°C, the cellular subgrains have disappeared and new subgrains with irregular shapes have formed which are bigger than 1 µm and are still confined inside the macro-grains. This continues until at very high temperatures (greater than 1200°C) the subgrains have grown into the size of the macro-grains and from this point on the macro-grains will start to grow and abnormal grain growth can possibly occur.

The critical alloying elements in 316 L are Cr, Ni and Mo. As mentioned previously, Cr and Mo are enriched at subgrain boundaries. As reported by Divya *et al*. [17], at high temperatures (greater than 1100°C), the diffusivity of Mo is 1.04 × 10^−14^ m^2^ S^−1^, which is almost the same as the diffusivity of Ni, which is 1.30 × 10^−14^ m^2^ S^−1^, and the activation energy for Mo is less than 300 kJ mol^−1^ [17] while for Ni it is approximately 5000 kJ mol^−1^ [18]. Therefore, at high temperatures (greater than 1100°C), Mo starts to diffuse from the subgrain boundaries and the subgrain boundaries merge together with the adjacent subgrain boundaries. This makes the subgrains grow larger while being confined in the macro-grains. Moreover, as mentioned before, dislocation pile-up occurs at the subgrain boundaries where Mo and Cr are segregated [11]. The segregated Mo and Cr prohibit the movement of dislocations and cause dislocation trapping. Dislocation trapping, along the cellular walls enriched by Cr and Mo, has been reported by Wang *et al*. [12]. As soon as Mo and Cr start to diffuse, dislocations annihilate by climb/glide and result in the growth of subgrains. It is suggested that the phenomenon of merging of the subgrains and growth dominates compared with the growth of the initial larger macro-sized grains. This can be explained by two facts. If the driving force for grain boundary migration is *P*, as the free energy reduction per unit area per unit distance, then the driving force is the free energy reduction associated with a unit volume of atoms detached from one grain and attached to another. Therefore, as expressed below, the driving force is the product of the grain boundary energy density and the curvature, where *γ* is the energy density and *K* is the curvature [19],

3.1 P=γK.

The area of the boundaries for the subgrains is larger than that of the boundaries for the irregular bigger grains (noting that the subgrain size is approx. 1 µm and the grains are approx. 10–25 µm). The subgrains (cells) have more curvature than the irregular grains. The curvature grain/subgrain growth mechanism has been confirmed by experiments and theoretical analysis [20]. On this basis, curvature-driven grain/subgrain growth has been successfully simulated quantitatively by the cellular automaton method [21]. The misorientation between the subgrains inside a bigger grain is smaller than that between the bigger grains themselves. A smaller misorientation between the subgrains, a larger curvature, and a high temperature enabling Mo diffusion and dislocation glide would lead to subgrains merging with adjacent subgrains (caused by dislocation annihilation and subgrain boundary deletion) and eventually will cause enlargement of the subgrains. Further growth of the subgrains is followed by the migration of these high-angle boundaries [22].

The ASTM grain size for samples thermally treated at 1100°C, 1200°C and 1400°C and the average grain size is calculated and expressed as follows [23]:

3.2 $$N_{m}{\left(M/100\right)}^{2}=2n-1$$

and

3.3 $$D(\mu m)=2^{\left(8.49-n/2\right)},$$

where *N*_m_ is the number of grains per square inch at magnification *M* and *n* is the ASTM grain size. The ASTM grain number and average grain size of samples thermally treated at 1100°C and 1200°C are listed in table 1 and compared with conventional 316 L treated at 1100°C (C-1100°C) and 1200°C (C-1200°C), respectively.

**Table 1.** ASTM grain number and average grain size for samples treated at 1100°C, 1200°C and 1400°C.

type (°C)	ASTM (*n*)	average grain size (µm)
SLM-1100°C	9–12	5–16
SLM-1200°C	7	32
SLM-1400°C	6	approx. 45
C-316 L at 1100°C [24]	5.5	approx. 50
C-316 L at 1200°C [24]	4.5	approx. 80

As seen from table 1, the grain size increases with heat treatment temperature. At 1100°C and 1200°C, the sizes of the grains are approximately 5–16 µm and 32 µm, respectively, which are much smaller than the grain sizes of conventional 316 L steel heat treated to the same temperatures, being 50 µm and 80 µm, respectively [24]. Consequently, it is expected that the tensile strength of SLM 316 L at 1100°C and 1200°C would be higher than that of conventional 316 L. To verify this, high-temperature tensile tests at 1100°C (SLM-1100°C) and 1200°C (SLM-1200°C) were performed, and the results were compared with those for conventional 316 L treated at 1100°C (C-1100°C) and 1200°C (C-1200°C) obtained from the literature [25]. The data from the mechanical tests are listed in table 2. The SLM-1100°C and SLM-1200°C are stronger than C-1100°C and C-1200°C. There are three possible reasons for this: (i) a smaller average grain diameter for SLM-1100°C and SLM-1200°C—15 µm and 32 µm, respectively, compared with 45 µm and 80 µm for C-1100°C and C-1200°C; (ii) the presence of a ferrite phase in the SLM samples thermally treated at 1100°C and 1200°C; and (iii) nucleation and formation of submicrometre sigma precipitates, which induce a strengthening effect. Slight differences in Mo and Cr and the heat treatment temperature of the laser-melted 316 L samples can cause sigma nucleation and growth. Moreover, it can be seen that the percentage of sigma precipitates decreases from 1100°C to 1200°C (shown in figure 1*a*) as they dissolve in the matrix [26]. Increased dislocation annihilation and a smaller amount of sigma precipitates are reported to be the plausible reason behind the reduction in strength from 300 MPa in SLM-1100°C to 150 MPa in SLM-1200°C. However, further detailed microstructure analysis with TEM is currently ongoing, which will be a follow-up work and the subject of an individual study.

**Table 2.** Mechanical properties of samples treated at 1100°C and 1200°C.

type (°C)	tensile strength (MPa)	elongation (%)
SLM-1100°C	300	15–18
SLM-1200°C	150	20
C-1100°C [25]	35–40	40
C-1200°C [25]	20	45

## 4. Conclusion

Subgrain boundaries merging and gradual growth of the subgrains restricted the growth of the initial grains. The continuous growth of the micrometre-sized subgrains instead of the macro-grains up to 1200°C resulted in a smaller average grain size (32 µm) than in conventionally made 316 L at the same temperatures. Inhibition of the piled-up dislocation movement at the subgrain boundaries caused by Mo, up to high temperatures, restricted microstructure coarsening and rapid grain growth. High temperatures greater than 1100°C enabled Mo (with high diffusivity and a low diffusion barrier), which is enriched at the subgrain boundaries, to diffuse. The formation of the sigma and ferrite phase, controlled grain growth and a smaller grain size in SLM 316 L resulted in a higher tensile strength at 1100°C and 1200°C than in conventionally made 316 L at the same temperatures. This positively and potentially can expand the application criteria and functionality of the 316 L material under high temperatures.

## Data accessibility

All data are presented within the paper.

## References

[1] Li JCM 2000 Microstructure and properties of materials, vol. 2. Singapore: World Scientific.

[2] Penn SJ, Alford NM, Templeton A, Wang X, Xu M, Reece M, Scharp K 1997 Effect of porosity and grain size on the microwave dielectric properties of sintered alumina. J. Am. Ceram. Soc. 80, 1885–1888. (doi:10.1111/j.1151-2916.1997.tb03066.x)

[3] Davidson KP, Singamneni S 2017 Magnetic characterization of selective laser melted Saf 2507 Duplex stainless steel. JOM 69, 569–574. (doi:10.1007/s11837-016-2193-6)

[4] Dehoff RT 1999 Engineering of microstructures. Mater. Res. 2, 111–126. (doi:10.1590/S1516-14391999000300002)

[5] Atkinson HV 1988 Overview no. 65: theories of normal grain growth in pure single phase systems. Acta Metall. 36, 469–491. (doi:10.1016/0001-6160(88)90079-X)

[6] Khu C 1964 Direct observation of annealing in crystals of F + 3% Si in the electron microscope. In Direct observation of imperfections in crystals (eds JB Newkirk, JH Wernick). New York, NY: Interscience.

[7] Dennis J, Bate PS, Humphreys JF 2007 Abnormal grain growth in metals. Mater. Sci. Forum 558–559, 717–722.

[8] Saeidi K, Gao X, Lofaj F, Kvetkova L, Shen Z 2015 Transformation of austenite to duplex austenite-ferrite assembly in annealed 316 L stainless steel consolidated by laser melting. J. Alloys Compd. 633, 463–469. (doi:10.1016/j.jallcom.2015.01.249)

[9] Zhong Y, Liu L, Wikman S, Cui D, Shen Z 2016 Intragranular cellular segregation network structure strengthening 316 L stainless steel prepared by selective laser melting. J. Nucl. Mater. 470, 170–178. (doi:10.1016/j.jnucmat.2015.12.034)

[10] Saeidi K, Niekter M, Olsen J, Shen Z, Akhtar F 2017 316 L designed to withstand intermediate temperature. Mater. Des. 135, 1–8. (doi:10.1016/j.matdes.2017.08.072)

[11] Saeidi K, Gao X, Zhong Y, Shen Z 2015 Hardened austenite steel with columnar sub-grain structure formed by laser melting. J. Mater. Sci. Eng. A 625, 221–229. (doi:10.1016/j.msea.2014.12.018)

[12] Wang YM et al. 2017 Additively manufactured hierarchical stainless steel with high strength and ductility. Nat. Mater. 17, 63–71. (doi:10.1038/NMAT5021) 29115290 10.1038/nmat5021

[13] Lysne VH 2016 Microstructural and physical investigation on the effect of the process parameters on stainless steel 316 L prepared by selective laser melting. Masters thesis, Faculty of Science and Technology, Prince of Songkla University, Thailand.

[14] Bertoli US, Guss G, Wu S, Matthews MJ, Schoenung JM 2017 In-situ characterization of laser-powder interaction and cooling rates through high-speed imaging of powder bed fusion additive manufacturing. Mater. Des. 135, 385–396. (doi:10.1016/j.matdes.2017.09.044)

[15] Zhongji S, Xipeng T, Shu BT, Wai YY 2016 Selective laser melting of stainless steel 316 L with low porosity and high build rates. Mater. Des. 104, 197–204. (doi:10.1016/j.matdes.2016.05.035)

[16] Niendorf T, Leuders S, Riemer A, Ricjard HA, Troster T, Schwarze D 2013 Highly anisotropic steel processed by selective laser melting. Metall. Mater. Trans. B 44, 794–796. (doi:10.1007/s11663-013-9875-z)

[17] Divya VD, Balam SSK, Ramamurty U, Paul A 2010 Interdiffusion in the Ni-Mo system. Scr. Mater. 62, 162–164. (doi:10.1016/j.scriptamat.2010.01.008)

[18] Huang KC, Shieu FS, Hsiao YH, Liu CY 2012 Ni interdiffusion coefficient and activation energy in Cu6Sn5. J. Electron. Mater. 41, 172–175. (doi:10.1007/s11664-011-1821-8)

[19] Suo Z 2004 Evolving small structures. Lecture 10: Grain growth. Princeton, NJ, USA. See http://imechanica.org/files/L10%20grain%20growth.pdf.

[20] Demirel M, Kuprat M, George D, Rollett A 2003 Bridging simulations and experiments in microstructure evolution. Phys. Rev. Lett. 90, 016106 (doi:10.1103/PhysRevLett.90.016106) 12570632 10.1103/PhysRevLett.90.016106

[21] Han F, Tang B, Kou H, Li J, Feng Y 2013 Cellular automata modeling of static recrystallization based on the curvature driven subgrain growth mechanism. J. Mater. Sci. 48, 7142–7152. (doi:10.1007/s10853-013-7530-3)

[22] Zhak KM, Pogrebnoi EN 1974 Mechanism of crystal growth in ferrite with silicon. Met. Sci. Heat Treat. 16, 300–303. (doi:10.1007/BF00679220)

[23] Pierson B 2012 Comparison of the ASTM comparative chart method and the mean line intercept method in determining the effect of solidification rate on the yield strength of AA5182. School of Engineering, Grand Valley State University, Allendale, MI, USA. See http://www2.gvsu.edu/peirsonb/solidification_yield_strength.pdf.

[24] Stanley JK, Perrotta AJ 1969 Grain growth in austenitic stainless steels. Metallography 2, 349–362. (doi:10.1016/0026-0800(69)90065-2)

[25] Gibbs TW, Wyatt HW 1960 Short time properties of type 316 stainless steel at very high temperatures. J. Basic Eng. 48, 481–488. (doi:10.1115/1.3662240)

[26] Sopousek J, Kruml T 1996 Sigma phase equilibria and nucleation in Fe-Cr-Ni alloys at high temperature. Scr. Mater. 35, 689–693. (doi:10.1016/1359-6462(96)00202-3)

